# A Phenotypic Characterization of Two Isolates of a Multidrug-Resistant Outbreak Strain of *Mycobacterium tuberculosis* with Opposite Epidemiological Fitness

**DOI:** 10.1155/2020/4741237

**Published:** 2020-04-08

**Authors:** Jinlong Bei, María Mercedes Bigi, Analía Lima, Qi Zhang, Federico Carlos Blanco, Beatriz Lopez, Ting Yu, Zhilin Wang, Zhangyan Dai, Zhuang Chen, Angel Adrian Cataldi, María del Carmen Sasiain, Viviana Ritacco, Silvia De la Barrera, Marcelo Abel Soria, Rosario Durán, Fabiana Bigi

**Affiliations:** ^1^AGRO-Biological Gene Research Center, Guangdong Academy of Agricultural Sciences (GDAAS), Guangzhou, China; ^2^University of Buenos Aires (UBA), School of Agronomy, Facultad de Agronomía (Universidad de Buenos Aires, Facultad de Agronomía), Buenos Aires, Argentina; ^3^Institut Pasteur de Montevideo & Instituto de Investigaciones Biológicas Clemente Estable, Uruguay; ^4^Institute of Biotechnology, National Institute of Agricultural Technology (INTA)/IABIMO-CONICET (Instituto de Biotecnología, Instituto Nacional de Tecnología Agropecuaria), Argentina; ^5^National Institute of Infectious Diseases ANLIS Carlos Malbrán (Instituto Nacional de Enfermedades Infecciosas-ANLIS Carlos Malbrán), Buenos Aires, Argentina; ^6^IMEX-CONICET, National Academy of Medicine (IMEX-CONICET, Academia Nacional de Medicina), Buenos Aires, Argentina

## Abstract

Tuberculosis (TB) is an infectious disease, caused by *Mycobacterium tuberculosis*, primarily affecting the lungs. The *M. tuberculosis* strain of the Haarlem family named M was responsible for a large multidrug-resistant TB (MDR-TB) outbreak in Buenos Aires. This outbreak started in the early 1990s and in the mid 2000s still accounted for 29% of all MDR-TB cases in Argentina. By contrast, a clonal variant of strain M, named 410, has caused a single tuberculosis case since the onset of the outbreak. The molecular bases of the high epidemiological fitness of the M strain remain unclear. To assess its unique molecular properties, herein, we performed a comparative protein and lipid analysis of a representative clone of the M strain (Mp) and the nonprosperous M variant 410. We also evaluated their growth in low pH. The variant 410 had higher levels of latency proteins under standard conditions and delayed growth at low pH, suggesting that it is more sensitive to stress stimuli than Mp. Moreover, Mp showed higher levels of mycolic acids covalently attached to the cell wall and lower accumulation of free mycolic acids in the outer layer than the 410 strain. The low expression of latency proteins together with the reduced content of surface mycolic acids may facilitate Mp to evade the host immune responses.

## 1. Introduction

*Mycobacterium tuberculosis*, the causative agent of tuberculosis (TB), achieves infection by deploying strategies that involve bacterial uptake and replication in host macrophages and by weakening or modulating host immune responses. TB is still one of the leading causes of mortality worldwide. Factors contributing to this situation are the HIV/AIDS pandemic, the deterioration of public health systems in developing countries and the emergence of multidrug-resistant (MDR) and extremely drug-resistant (XDR) forms of TB. In 2019, the World Health Organization (WHO) reported that 3.4% of new TB cases and 18% of previously treated cases had MDR-TB or rifampicin-resistant TB (RR-TB) (https://apps.who.int/iris/bitstream/handle/10665/329368/9789241565714-eng.pdf?ua=1).

The *M. tuberculosis* strain named M, which belongs to the Haarlem family, was responsible for a large and prolonged MDR-TB outbreak in Buenos Aires that started in the early 1990s in a referral hospital for infectious diseases [1]. Thereafter, the M strain has disseminated into the community, and between 2003 and 2009, it was still responsible for 29% of all MDR-TB cases in Argentina [2]. This strain has only recently started to decline [3]. By contrast, its clonal variant 410 has caused a single tuberculosis case since the onset of the outbreak [4].

Increasing evidence has shown that the M strain can manipulate host immune responses for its own benefit. M induces nonfunctional CD8+ cytotoxic T cell activity, whereas its variant 410 induces a cytotoxic response comparable to that of the strain H37Rv [5, 6]. The cytotoxic T cell activity is associated with lysis of *M. tuberculosis*-infected macrophages [7] and, as a consequence, reduced *M. tuberculosis* viability [8]. The poor cytotoxic activity induced in M infection may allow the bacteria to evade the immune system and persist in the hosts.

In monocyte-derived macrophages, M grows more slowly and induces lower levels of TNF-*α* and IL-10 than 410. By contrast, in the human promonocytic U937 cell line, both M and 410 display similar growth patterns [4]. Strain M, however, induces a significantly higher proinflammatory response than 410. Thus, its interaction with the host cells is not sufficient to explain the high epidemiological success of the strain M.

Recently, we searched for all polymorphisms occurring between an early representative of the M prototype strain, hereafter named Mp, and its contemporary variant 410. In that study, polymorphisms located in 11 proteins and five intergenic regions were identified [9]. Some of these polymorphisms were located in proteins or promoter regions involved in the metabolism of cell wall components, whereas others occurred in proteins associated with drug resistance [9].

Some progress has been made in the understanding of the molecular mechanisms that determine the epidemiological success of certain *M. tuberculosis* lineages and of the M family in particular. The molecular bases of the high epidemiological fitness of strain Mp, however, remain unclear.

The objective of this study was to shed light on the molecular bases that sustain the epidemiological success of Mp. For this purpose, we followed a proteomic approach by comparing the complete cellular proteome of both strains and performed a lipid analysis by thin layer chromatography.

This work shows evidence that relates the transmission capacity of a MDR Haarlem strain of *M. tuberculosis* with the accumulation of stress proteins and the distribution of mycolic acids in the subcomponents of the cell wall.

## 2. Methods

### 2.1. *M. tuberculosis* Isolates

The *M. tuberculosis* Mp and 410 strains belong to the Haarlem H2 genotype (SIT 2, SNP-barcode sublineage 4.1.2.1). The strains are resistant to pyrazinamide, streptomycin, isoniazid, and rifampicin. Mp is also resistant to kanamycin and ethambutol. Both strains are genetically close, since they have similar molecular markers [9].

### 2.2. Protein Extraction, Digestion, and TMT Labelling

The *M. tuberculosis* strains were grown for 45 days without agitation in Middlebrook 7H9 supplemented with 0.4% glycerol, 0.5% bovine albumin, and 0.4% glucose without Tween 80 in sealed T75 flasks at 37°C. The bacterial cells were centrifuged at 1,000 × g for 20 min at 4°C. The pellets were washed with buffer phosphate saline, resuspended in ice-cold lysis buffer (50 mM Tris-HCl, pH 7.5, 25 mM NaCl, 5 mM DTT, and 1 mM PMSF), and transferred to a 2 ml screw cap microcentrifuge tube containing 0.1 mm glass beads (Sigma-Aldrich). The cells were disrupted with a Precellys 24 homogenizer (Bertin Instruments) by three cycles of the bead beater for 20 s at 6 m s^−1^. The homogenates were centrifuged at 10,000 × g for 10 min before adding ice-cold lysis buffer 2 (7 M urea, 2 M thiocarbamide, 1% CHAPS, and protease inhibitor) to the resulting pellets. The supernatants were then precipitated with three volumes of ice-cold acetone and incubated at -20°C for 4 h. The protein pellets were resuspended in a buffer (200 mM tetraethylammonium bromide, pH 8.5, 8 M urea, and 2 M thiocarbamide). Protein concentrations were determined by the Bradford assay using bovine serum albumin as a standard. Triplicates of each strain were used for the proteomic analysis.

A total of 100 *μ*g proteins was reduced with 200 mM Tris(2-carboxyethyl)phosphine (TCEP) at 37°C for 4 h and alkylated for 30 min in the dark with 375 mM iodoacetamide. Then, six volumes of prechilled acetone were added and incubated overnight at -20°C. The protein pellets were resuspended in 100 mM tetraethylammonium bromide. Subsequently, the samples were digested using trypsin (Promega) at a ratio of 1 : 50 overnight at 37°C. The digested peptides were labelled with 6-plex TMT reagents according to the manufacturer's instructions (Thermo Scientific). The TMT labelling efficiency was assessed by Orbitrap Fusion, and then the samples were combined and fractionated by high pH reversed-phase (hpRP) fractionation as described below.

### 2.3. High pH Reversed-Phase Fractionation

The mixed TMT labelled peptides were desalted with Sep-Pak SPE cartridges (Waters) for subsequent hpRP separation in a Dionex UltiMate 3000 model as described previously [10]. Finally, 48 collected fractions were merged into 12 mixtures (Supplementary figure 1).

### 2.4. Nano-LC-MS/MS Analysis by Orbitrap Fusion

The nano-LC-MS/MS analysis was carried out on an Orbitrap Fusion Tribrid (Thermo Fisher Scientific, San Jose, CA) mass spectrometer connected to an UltiMate 3000 RSLCnano System (Dionex, Thermo Fisher, Sunnyvale, CA). All mixtures were reconstituted in 2% acetonitrile and 0.1% formic acid. Each reconstituted mixture was loaded on a PepMap C18 trapping column (3 *μ*m, 75 *μ*m × 2 cm, Dionex) at 6 *μ*l/min, and peptides were separated on a PepMap C18 RP analytical column (2 *μ*m, 50 *μ*m × 15 cm, Dionex) at 300 nl/min. A 163 min gradient was used as follows: 2-6% buffer B (100% ACN and 0.1% formic acid) for 5 min; 6-22% buffer B, 100 min; 22-35% buffer B, 30 min; 35-90% buffer B, 5 min; 90% buffer B, 10 min; 90-2% buffer B, 2 min; and 2% buffer B, 11 min, before returning to buffer A (2% ACN and 0.1% formic acid).

The separated peptides were analysed on the Orbitrap Fusion with a nanospray voltage of 1.9 kV and a source temperature of 275°C in the positive ion mode. Full MS spectra were acquired on the Orbitrap at a resolution of 120,000, across a mass range of 350 to 1,550 *m*/*z*. The automatic gain control target of MS1 was set to 2 × 10^5^ with a max injection time of 50 ms. Tandem mass spectra (with an automatic gain control target of 1 × 10^5^ and a maximum injection time of 80 ms) were recorded on the Orbitrap at a resolution of 30,000 and made by HCD at a normalized collision energy of 40. The top 10 most intense peaks were selected for fragmentation in each cycle of the data-dependent mode. All data were acquired with Xcalibur 3.1 software (Thermo Fisher Scientific).

### 2.5. Protein Identification and Quantitation

Data analysis was carried out using PatternLab for Proteomics software (version 4.1.1.15) [11]. A target-decoy database from *M. tuberculosis* H37Rv sequences downloaded from UniProt (March 2019) (https://www.uniprot.org), with the inclusion of the 127 most common mass spectrometry contaminants, was generated for the analysis. The raw files were searched against the *M. tuberculosis* database using the Comet search engine [12] and applying the following search parameters: trypsin as a proteolytic enzyme with full specificity, methionine oxidation and asparagine/glutamine deamidation as the variable modification, and cysteine carbamidomethylation and lysine 6-plex TMT modification (including N-terminus) as the fixed modification. The precursor ion tolerance was set at 40 ppm, and a clear *m*/*z* range was specified from 127 to 131.

The peptide spectrum matches were filtered using PatternLab's Search Engine Processor (SEPro), including MS2 in the results. An acceptable false discovery rate (FDR) criterion was set at 1% of the protein level [13]. Results were processed to filter out peptides with more than six amino acid residues and proteins with a minimum of two spectrum matches.

The PatternLab for Proteomics isobaric analyser module was used to analyse the data quantitatively. YADA software [14] was employed with its default configuration on the MS1 and MS2 extracted files. A peptide quantitation report was generated using SEPro and YADA files and by setting TMT-6 as the label according to instructions described previously [11].

### 2.6. Overrepresentation Test

A statistical overrepresentation test of GO biological processes (release 20190606) was performed using the Panther Server (http://pantherdb.org). The release date of the GO dataset was 2019-02-02. The analysis was performed with the TubercuList gene identifiers using the *M. tuberculosis* database as a reference list. Fisher's exact test type in combination with the calculated discovery rate correction was used for the analysis [15].

### 2.7. Gene Expression

The RNA extractions were performed as follows: Cultures of Mp and 410 were grown without agitation in Middlebrook 7H9 supplemented with 0.4% glycerol, 0.5% bovine albumin, 0.4% glucose, and 0.05% Tween 80 for 45-60 days at 37°C. The cultures were centrifuged, and cell pellets were immediately resuspended in 1 ml of TRIzol (Sigma-Aldrich) and transferred to a 2 ml screw cap microcentrifuge tube containing 0.1 mm silica glass beads (Sigma-Aldrich). The cells were disrupted with a Precellys 24 homogenizer (Bertin Instruments) by three cycles of the bead beater for 20 s at 6 m s^−1^, and total RNA was purified as previously described [16]. The integrity of total RNA was assessed in a 0.8% agarose gel (Supplementary figure 2). Finally, the RNA samples were treated with Ambion DNase I (Life Technologies) following the manufacturer's specifications.

RT-qPCR were performed as previously described [17] with specific primers (Supplementary table 3). All qPCR were performed in duplicate, and the average values of duplicates were analysed using the LinRegPCR software [18] as previously described [16]. The statistical analyses were done with the *fg* statistic software [19].

### 2.8. Lipid Analysis

Total lipids from bacterial cells grown until the stationary phase (OD600_nm_ 0.8-1) in Middlebrook 7H9 supplemented with 0.5% glycerol, 0.5% bovine albumin, and 0.4% glucose without agitation were extracted following procedures described earlier [20]. Fatty acid and mycolic acid methyl esters (FAMEs and MAMEs, respectively) were derived from extractable lipids and delipided cells (cells depleted from extractable lipids) as described by Stadthagen et al. [20] and analysed by thin layer chromatography (TLC) on silica gel 60F254 by loading 0.25 mgr of lipids per lane. TLCs were developed using *η*-hexane : ethyl acetate (95 : 5 *v*/*v*) (thrice) and revealed by spraying with a CuSO_4_-phosphoric acid solution and heating.

## 3. Results

### 3.1. In Vitro Growth of Mp and 410 Strains

**Figure 1:**
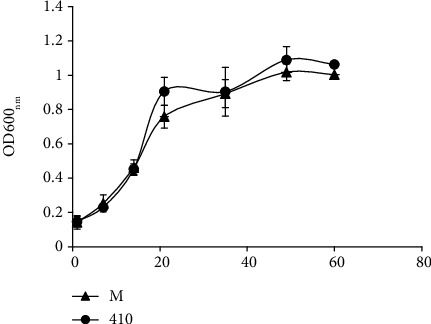
*In vitro* growth of Mp and 410 strains. The evaluated *M. tuberculosis* strains were grown in Middlebrook 7H9 supplemented with 0.5% glycerol, 0.5% bovine albumin, 0.4% glucose, and 0.05% Tween 80. The bacterial growth was determined by OD600_nm_. Data represent the mean ± SD of three independent replicates.

We first assessed the *in vitro* growth of the Mp and 410 strains by measuring the optical density (OD) at 600 nm of cultures incubated without agitation in the presence of the detergent Tween 80. At 45-50 days of culturing, both strains entered the stationary phase (Figure 1), indicating that in this *in vitro* condition of growth, Mp and 410 had similar fitness. The oxygen availability assays revealed low content of oxygen in the bacterial cultures at day 60, as evidenced by the complete disappearance of blue coloration at this point (methylene blue) (data not shown). These results indicate that at day 60, cultures of the Mp and 410 strains are in hypoxia.

### 3.2. Proteomic Analysis

We used a TMT-6plex labelling strategy associated with two-dimensional liquid chromatography and mass spectrometry in tandem to compare the total cellular proteomes of the strains Mp and 410. We obtained total cellular proteins from static cell cultures in the early stationary phase growth in sealed flasks. Detergents were omitted from the cultures to preserve the cell surface protein content. For the proteomic analysis, we analysed three biological replicates per strain; each replicate consisted of a mix of two batch cultures. Therefore, we analysed six independent batch cultures for each strain.

The identified proteins covered ∼69% of the predicted *M. tuberculosis* proteome (Supplementary table 1). The proteomic analysis indicated a high level of similarity between 410 and Mp when grown *in vitro* until the stationary phase (Supplementary table 1), since only 12 of the differentially accumulated proteins showed a fold change (FC) greater than |2| and a *P* value below 0.01 between the strains (Table 1). One of the most accumulated proteins in the strain 410 was IdsA. This result is in accordance with the results of our previous study, in which this protein, a geranylgeranyl pyrophosphate synthetase protein likely involved in lipid biosynthesis, showed higher levels of transcription in 410 than in Mp [9]. The strain 410 also accumulated three proteins from the DevR regulon (Rv0569, Rv2031c, and Rv2007c), two proteins of unknown functions (Rv3221c and Rv2081c), a GCN5-related N-acetyltransferase (Eis, Rv2416c), and a putative ribonuclease E (Rv2444c).

Rv2057c, Rv2058c, Rv2154c, and Rv0290 showed a higher accumulation in Mp. Rv2057c and Rv2058c are ribosomal proteins, whereas Rv2154c is the peptidoglycan glycosyltransferase FtsW, which seems to participate in the peptidoglycan synthesis [21]. On the other hand, Rv0290 is an ESX-3 secretion system protein.

A global enrichment analysis considering all differentially accumulated proteins (FC > ∣1.3∣ and pVal < 0.03) showed the protein families that distinguished both strains. Whereas Mp accumulated proteins associated with active metabolism, 410 had more protein families related to stress and hypoxia (Table 2 and Supplementary table 2).

### 3.3. Transcriptional Analysis of the DevR Regulon

DevR is the response regulator of a two-component system, which is activated in response to hypoxia and upon exposure to a number of stresses [22].

Because the proteomic results showed higher abundance of stress proteins in the 410 strain, we next measured the transcriptional level of the DevR-regulated proteins *Rv0569*, *Rv2007c*, and *hspX* at the early and late exponential phases. We also assessed the transcription level of *Rv1738c*, because this gene encodes a key component of a stress response system [23] and because its FC was close to the cut-off established in the proteomic analysis (FC 1.990).

In the early stationary (OD600_nm_ ~ 0.8) growth phase, the strain 410 showed a significant upregulation of the transcription of all genes except *Rv0569* (Supplementary figure 3A) in comparison to Mp. In the late stationary (OD600_nm_ ~ 1) growth phase, on the other hand, *Rv2007c*, *hspX*, and *Rv0569* were upregulated in the strain Mp; *Rv1738* showed no differences between strains (Supplementary figure 3B).

### 3.4. Response to Acid Stress

**Figure 2:**
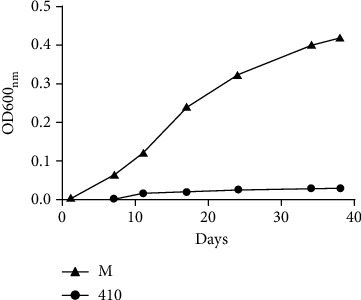
*In vitro* growth of Mp and 410 strains at low pH. The assessed *M. tuberculosis* strains were grown in Middlebrook 7H9 supplemented with 0.5% glycerol, 0.5% bovine albumin, 0.4% glucose, and 0.05% Tween 80 at pH 5.7. The bacterial growth was determined by OD600_nm_ measurements. One experiment of three with similar results is shown in the figure.

The dormancy or latency program, in which the DevR regulon plays a central role, is a survival mechanism that allows mycobacteria to resist unfavourable conditions without replication. This program is activated in response to a variety of stresses, such as starvation, hypoxia, and low pH. To determine if the latent phenotype of the 410 strain is a consequence of a greater sensitivity to stress conditions in comparison to the Mp strain, we evaluated the growth of Mp and 410 at pH 5.7. The replication of 410 was delayed compared to that of Mp (Figure 2), suggesting that 410 rapidly enters into a nonreplicative state under unfavourable conditions.

### 3.5. Lipid Analysis

The proteomic analysis showed significant variation between the Mp and 410 strains in the content of enzymes involved in mycolic acid biosynthesis (Table 2). Mycolic acids are very long-chain *α*-alkyl *β*-hydroxy fatty acids present in the cell wall of the Mycolata taxon. In *M. tuberculosis*, mycolic acids are similar in length but may harbour a variety of functional groups (such as double binds, cyclopropanations (*cis* or *trans*), keto, and methoxy groups) that give rise to a number of subfamilies [24]. They can be covalently attached to the cell wall arabinogalactan or can be esterified to trehalose to produce trehalose monomycolate (TMM) and trehalose dimycolate (TDM) [25], or they can remain free. Given the importance of mycolic acids in the interaction of *M. tuberculosis* with the host [26], we subsequently assessed the mycolic acid content in both strains.

**Figure 3:**
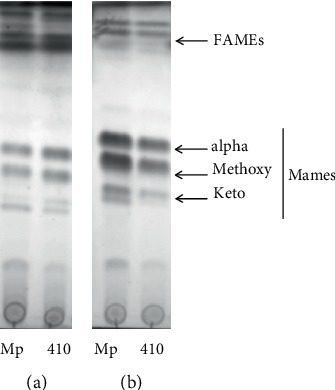
The strains 410 and Mp showed different content of mycolates. Fatty acid and mycolic acid methyl esters (FAMEs and MAMEs, respectively) were derived from extractable lipids (a) and delipided cells (b) of Mp and 410 strains cultured in 7H9 supplemented with 0.5% glycerol, 0.5% bovine albumin, and 0.4% glucose without agitation until reaching the stationary growth phase. TLC plates were developed in the solvent system *η*-hexane : ethyl acetate (95 : 5) (thrice) and revealed with CuSO_4_ and heating.

A TLC analysis of fatty acid methyl esters (FAMEs) and mycolic acid methyl esters (MAMEs) from the extractable cellular lipids showed an increase in the mycolic acid content of the strain 410 in comparison to the strain Mp (Figure 3(a) and Supplementary figure 4A). By contrast, the content of mycolic acids attached to the cell wall was higher in Mp than in 410 (Figure 3(b) and Supplementary figure 4B). These phenotypes were reproducible throughout more than three independent experiments.

Altogether, these results demonstrated that although the total abundance of mycolic acids is equivalent in both strains, their distribution in the outer and inner leaflets of the mycomembrane varies.

## 4. Discussion

In this study, we investigated the molecular mechanisms responsible for the differential transmissibility of the epidemic strain Mp and its sporadic variant 410. To this purpose, we determined the relative abundance of total cellular proteins of both strains grown until reaching the stationary phase without aeration by following a robust proteomic approach. The approach consisted of the simultaneous detection, in the same run, of the peptides expressed in all the samples and replicates, thus avoiding the experimental variability inherent to multiple runs.

This analysis retrieved nearly 62% of the *M. tuberculosis* proteome. In a previous study, Albrethsen et al. [27] have identified 1,176 proteins in a culture supernatant of *M. tuberculosis*. Considering that the secreted proteins were excluded in our study and that these subsets of proteins represent a high proportion of total proteins [27], we can conclude that the coverage in our proteomic analysis is appropriate.

In our study, the high level of genomic conservation between the strains Mp and 410 [9] was translated to similar proteomes for the Mp and 410 strains. Only 12 proteins showed higher accumulation (FC > 2) in one strain relative to the other. Most of the detected proteins differed by no more than 1.5-folds between the strains.

DevR is the response regulator of a two-component system, which is activated in response to hypoxia and nitric oxide by the histidine kinases DevS or DosT [22]. Boon and Dick [28] have reported that DevR is responsible for the dormancy/latent stage of *M. tuberculosis* and that upon mutation of *devR*, the bacteria fails to enter dormancy and thus dies in a Wayne culture system of hypoxia. Furthermore, the exposure of *M. tuberculosis* mutants lacking DevR to low oxygen tension induces the expression of more than 100 genes, of which 48 are under the control of DevR [22]. In this study, at the early stationary phase in the presence of detergent, the 410 strain showed higher transcription of key stress DevR genes (*Rv1738*, *Rv2007c*, and *hspX*) than the Mp strain under that condition, which is concordant with our proteomic results. By contrast, at the late stationary phase, the strain 410 downregulated transcription of the stress genes *Rv0569*, *Rv2007c*, and *hspX*. These results indicate that the timing of stress response varies between strains. Moreover, the delayed replication of the 410 strain at low pH supports the idea that this strain activates the DevR program more rapidly than the Mp strain under stress stimuli.

Altogether, these results suggest that Mp displays lower sensitivity to stress stimuli and remains metabolically active longer than the 410 strain. In accordance with this conclusion, Mp accumulated higher amounts of proteins involved in translation (Rv2058c and Rv2057c) and cell division (FstW), whereas 410 overexpressed Rv1738, a protein likely involved in the shutdown of the ribosomal protein synthesis of *M. tuberculosis* induced under nitrosative and hypoxia stresses [23].

In a previous study, we identified two proteins with S-adenosylmethionine- (SAM-) dependent methyltransferase domains, Rv3787c and CmaA2, as polymorphic between the 410 and Mp strains [9]. According to TBDB (http://genome.tbdb.org/), Rv0081 has a putative binding site in the promoter region of *Rv3787c*, which suggests that *Rv3787c* is part of the Rv0081 regulon. Interestingly, herein, the strain Mp accumulated less hypoxia-related proteins than the strain 410 under an oxygen-limited condition. Thus, the fact that Rv0081 is a hub regulator involved in the adaptation of *M. tuberculosis* to hypoxic condition [29] may connect the polymorphism of Rv3787c to a retarded response of Mp to hypoxia. Nevertheless, this hypothesis needs to be demonstrated with experimental evidence.

Mariotti et al. [30] have demonstrated that latent *M. tuberculosis* displays higher capacity to stimulate specific human T lymphocytes than replicative *M. tuberculosis*. In addition, Mp induced a poor activation of cytotoxic CD8+ T cells from healthy donors in *ex vivo* experiments, in comparison to 410 and H37Rv [5, 31]. Thus, the latent phenotype of 410 may impair its capacity of evading the immune system in relation to Mp. However, the role of latency in tuberculosis transmission is not completely clear. For instance, the DevR latent regulon of members of the W-Beijing family, which is a lineage with an epidemic spread, is constitutively upregulated [32]. Reed et al. proposed this phenomenon as an advantage to *M. tuberculosis* that allows perpetuation and rapid dissemination of the population. Conversely, Duncan et al. [33] showed that an *M. tuberculosis* natural mutant circulating among homeless people in Canada displayed decreased expression of the hypoxia-induced regulon DevR in relation to its isogenic wild-type strain. Therefore, this last study suggests that the lack of latency proteins does not affect the transmission of *M. tuberculosis*.

The TLC analysis showed increased mycolic acid transferred into the cell wall and the simultaneous lower abundance of free mycolic acids in the Mp strain in relation to the 410 strain. The reasons of this differential distribution of mycolic acids in Mp and 410 are still unclear. However, previous findings and the findings presented in the current study may explain in part this result. For instance, Telenti et al. have previously reported that a treatment of mycobacteria with ethambutol produced the opposed imbalance in mycolic acid distribution, with increased accumulation of free mycolates and reduced transfer of these lipids to the cell wall [34]. Ethambutol inhibits EmbB (Rv3795), which is an arabinosyltransferase involved in the synthesis of the mycobacterial cell wall arabinan [35]. Interestingly, Mp carries a mutation in *embB* [9] that may be associated with resistance to ethambutol. Thus, this mutation in Mp may improve the enzymatic activity of EmbB, increasing the availability of arabinogalactan for mycolylation.

*M. tuberculosis* has five S-adenosylmethionine- (SAM-) dependent methyltransferases that participate in the cyclopropanation of mycolic acids (MmaA2, MmaA1, CmaA1, CmaA2, and PcaA). Thus, polymorphisms in the methyltransferases Rv3787c and CamA2 may also contribute to the differential profile of mycolic acids in the 410 and Mp strains.

In addition, Dulberger et al. have proposed a model for mycobacterial cell wall remodelling in growth and stasis [26], in which free mycolic acids increase during stasis, while TDM, TMM, and mycolated arabinogalactan decrease or keep constant. According to this model, the 410 strain displayed a more “stasis” lipid profile relative to the Mp strain. In fact, these profiles are consistent with the proteomic results of this study. However, we did not detect a significant decrease in TDM and TMM content in the 410 strain in relation to the Mp strain in the TLC experiments (Supplementary figure 4C).

Substantial evidence demonstrates that modified mycolic acids play specific and diverse roles in *M. tuberculosis* pathogenesis [36, 37, 38]. Indeed, the complete lack of mycolate cyclopropanation suppresses the host immune response [39]. These previous observations suggest that the decrease in free mycolic acids in the strain Mp can also contribute to the lower immune response induced *ex vivo* by this strain in human cells [40].

Although the most distinctive feature of the strain Mp with respect to the variant 410 is its ability to perpetuate in the community, both strains are highly conserved at the genomic [9] and proteomic (this study) levels. Thus, the few differences detected in stress protein accumulation and mycolic acid localization between strains may play a central role in the epidemiological behaviour of *M. tuberculosis*. This hypothesis needs to be confronted with further experimental evidence from the study of other *M. tuberculosis* strains with different transmissibility.

## Figures and Tables

**Table 1:** Most differentially accumulated proteins between Mp and 410 strains.

Protein	Rv number	Log 2 fold change^a^	*P* value	Description
Rv3221c	Rv3221c	-2.78	8.20*E*-03	Biotinylated protein
IdsA2	Rv2173	-2.57	1.20*E*-03	Probable geranylgeranyl pyrophosphate synthetase
Rv0569	Rv0569	-2.55	1.90*E*-03	Uncharacterized protein
hspX	Rv2031c	-2.52	1.20*E*-09	alpha-Crystallin
fdxA	Rv2007c	-2.27	3.40*E*-04	Ferredoxin
Eis	Rv2416c	-2.19	6.70*E*-03	N-acetyltransferase Eis
Rv2081c	Rv2081c	-2.16	1.20*E*-02	Uncharacterized protein
Rne	Rv2444c	-2.06	3.90*E*-03	Possible ribonuclease E
RpmB2	Rv2058c	2.03	2.40*E*-03	50S ribosomal protein
FtsW	Rv2154c	2.07	1.00*E*-02	Probable peptidoglycan glycosyltransferase
EccDE	Rv0290	2.22	5.90*E*-03	ESX-3 secretion system protein
RpmG1	Rv2057c	2.77	5.10*E*-03	50S ribosomal protein

^a^Log 2 fold change in the protein relative abundance of the strain M with respect to the strain 410. Negative fold-change values indicate that proteins are overrepresented in the strain 410.

**(a):** 

Mp								
*Mycobacterium tuberculosis* (REF)		Text box input						
GO biological process complete	#	#	Expected	Fold enrichment	+/−	Raw *P* value	FDR	Genes

tRNA aminoacylation for protein translation	20	14	2.26	6.18	+	2.13*E*-06	1.32*E*-04	*pheT*, *asps*, *alas*, *SerS*, *tyrS*, *lysS1*, *proS*, *gltX*, *leuS*, *argS*, *hiss*, *metG*, *cysS*, *valS*
Mycolic acid biosynthetic process	12	7	1.36	5.15	+	1.82*E*-03	4.51*E*-02	*mmaA2*, *kasB*, *inhA*, *mmaA4*, *cmaA2*, *cmaA1*, *mmaA3*
Glutamine metabolic process	20	10	2.26	4.42	+	4.92*E*-04	1.60*E*-02	*glnA2*, *asnB*, *glnA4*, *gltB*, *cobQ*, *hisH*, *glmS*, *pyrG*, *pknG*, *guaA*
Tricarboxylic acid cycle	25	11	2.83	3.89	+	6.03*E*-04	1.85*E*-05	*icd2*, *gabD1*, *glcB*, *citA*, *gabD2*, *can*, *lpdC*, *sdhA*, *icd*, *sdhB*, *fumC*
Ribonucleoside monophosphate biosynthetic process	32	12	3.62	3.31	+	1.07*E*-03	3.02*E*-02	*purT*, *purH*, *pyrD*, *purD*, *purA*, *purB*, *add*, *purr*, *carB*, *guaA*, *purM*, *prs*
Ribonucleoside metabolic process	88	22	9.96	2.21	+	1.92*E*-03	4.54*E*-02	*relA*, *tesB2*, *purT*, *pyk*, *pfkA*, *purH*, *pyrD*, *gpmA*, *glcB*, *Rv0386*, *aceE*, *purD*, *coax*, *lpdC*, *purA*, *pyrG*, *fba*, *purB*, *purU*, *carB*, *guaA*, *purM*
Nucleotide biosynthetic process	93	23	10.53	2.18	+	1.50*E*-03	4.05*E*-02	*ppnK*, *nrdF2*, *cmk*, *relA*, *purT*, *dcd*, *nadD*, *purH*, *pyrD*, *nadE*, *Rv0386*, *nrdF1*, *Rv2488c*, *purD*, *coax*, *purA*, *pyrG*, *purB*, *purU*, *carB*, *guaA*, *purM*, *prs*

**(b):** 

410								
*Mycobacterium tuberculosis* (REF)		Text box input						
GO biological process complete	#	#	Expected	Fold enrichment	+/−	Raw *P* value	FDR	Genes

Protein folding	14	5	0.33	15.17	+	5.21*E*-05	1.62*E*-02	*groEL2*, *dnaK*, *grosS*, *grpE*, *hspX*
Response to hypoxia	46	15	1.08	13.85	+	2.54*E*-12	5.54*E*-09	*groEL2*, *fdxA*, *devR*, *Rv2623*, *hmp*, *icl*, *Rv0569*, *Rv2005c*, *narX*, *acg*, *Rv2629*, *hspX*, *Rv3134c*, *hrp1*
Positive regulation of cellular process	33	6	0.78	7.72	+	2.23*E*-04	4.86*E*-02	*dnaK*, *devR*, *phoP*, *lpqH*, *bpa*, *hbhA*
Response to host immune response	129	11	3.04	3.62	+	3.10*E*-04	6.14*E*-02	*fdxA*, *Eis*, *desA1*, *icl*, *lprA*, *acg*, *hspX*, *lpqH*, *sodC*, *Rv1738*, *hrp1*

## Data Availability

Data are available for research purposes.

## Supplementary Materials

Supplementary Materials Supplementary Material: Supplementary Table 1, Supplementary Table 2, and Supplementary Table 3. Supplementary Figure 1: (a) chromatogram profile of the high pH reversed-phase (hpRP) fractionation. (b) Schematic representation of the 48 fractions collected in the hpRP chromatography. The labels M1 to M12 in each well indicate the pattern of mixture. Supplementary Figure 2: total RNA obtained from cultures in the stationary phase (OD600_nm_ ~ 1) and resolved in 0.8% agarose gel. Supplementary Figure 3: comparative gene expression of selected DevR regulon genes between strains by RT-qPCR at (a) early stationary and (b) late stationary growth phases. Fold changes of Mp/410 strains were calculated using the expression levels of the sigA mRNA as a reference gene and the 410 variant as a calibrator. Data were analysed using a random permutation test (*fg* statistic software ^∗^*P* < 0.05). The bars represent average expression ratios of six samples ± SD between the Mp strain and the 410 variant. Supplementary Figure 4: FAMEs and MAMEs were derived from extractable lipids (a) and delipided cells (b) of the Mp and 410 strains. TLC plates were developed in the solvent system *η*-hexane : ethyl acetate (95 : 5) (thrice) and revealed with CuSO_4_ and heating. (c) Total extractable lipids of the Mp and 410 strains were analysed by TLC developed in the solvent system chloroform : methanol : water (90 : 10 : 1).
