# Supplementary figures and images for: A Phenotypic Characterization of Two Isolates of a Multidrug-Resistant Outbreak Strain of Mycobacterium tuberculosis with Opposite Epidemiological Fitness
Source: Biomed Res Int. 2020 Apr 8;2020:4741237. doi: 10.1155/2020/4741237 (PMC7168692; doi:10.1155/2020/4741237)

**A**


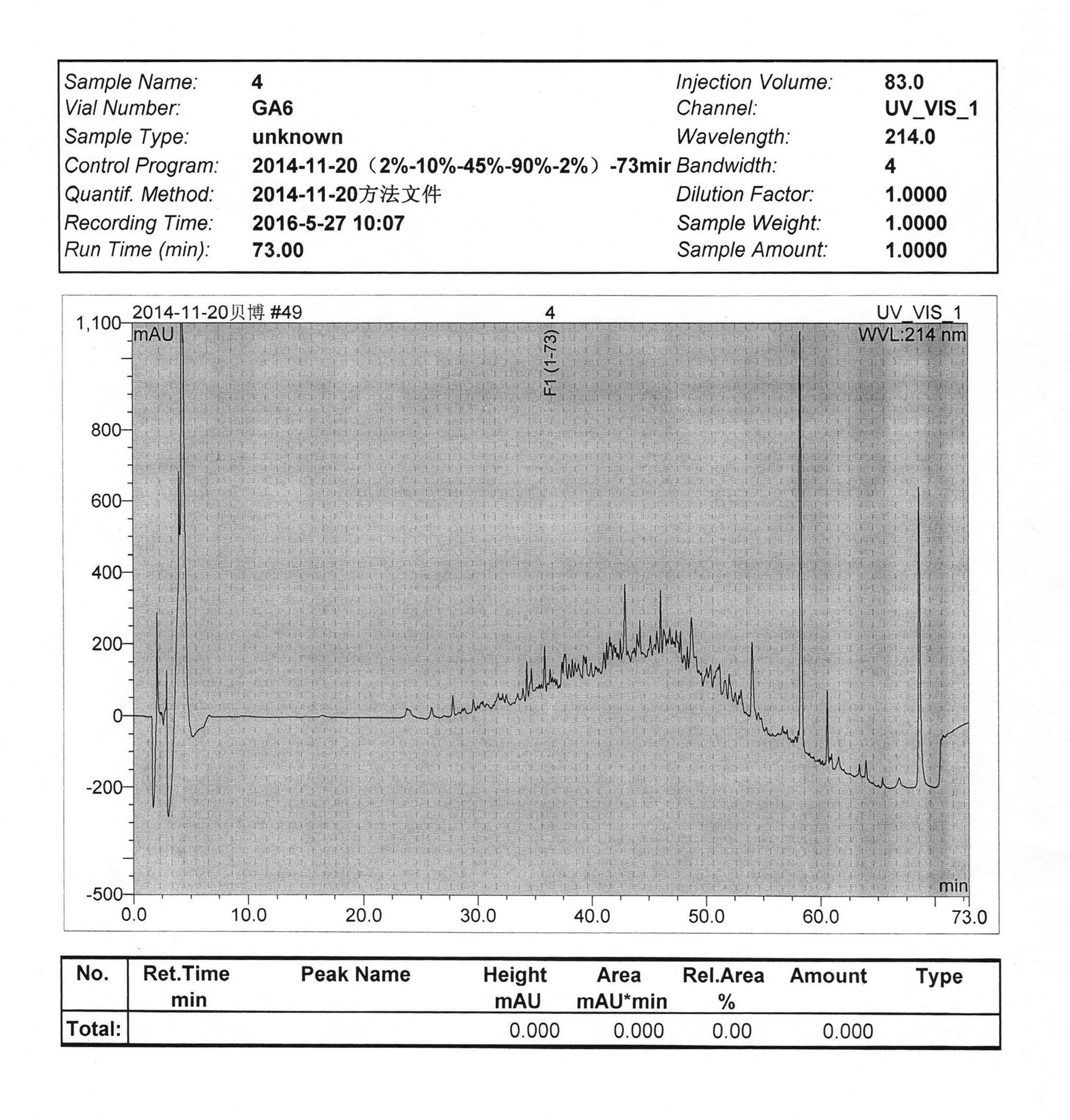


**B**


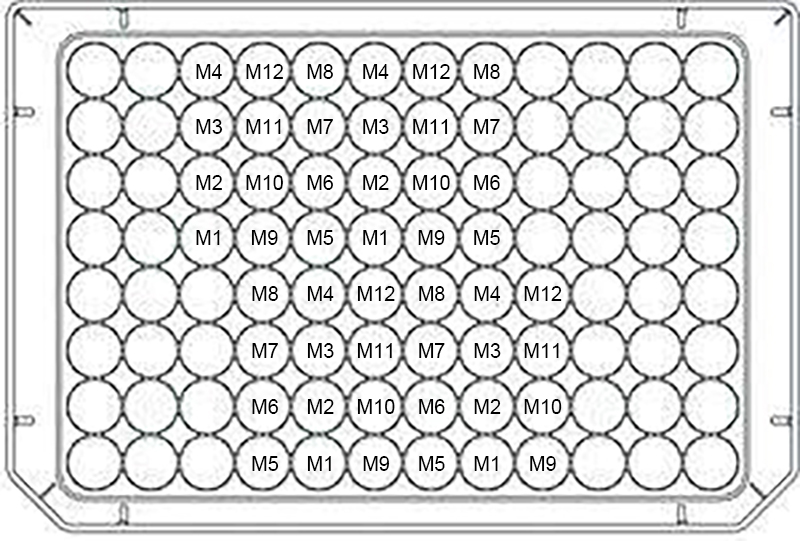

Supplement: Supplementary Materials — Supplementary Material: Supplementary Table 1, Supplementary Table 2, and Supplementary Table 3. Supplementary Figure 1: (a) chromatogram profile of the high pH reversed-phase (hpRP) fractionation. (b) Schematic representation of the 48 fractions collected in the hpRP chromatography. The labels M1 to M12 in each well indicate the pattern of mixture. Supplementary Figure 2: total RNA obtained from cultures in the stationary phase (OD600nm ~ 1) and resolved in 0.8% agarose gel. Supplementary Figure 3: comparative gene expression of selected DevR regulon genes between strains by RT-qPCR at (a) early stationary and (b) late stationary growth phases. Fold changes of Mp/410 strains were calculated using the expression levels of the sigA mRNA as a reference gene and the 410 variant as a calibrator. Data were analysed using a random permutation test (fg statistic software ∗P < 0.05). The bars represent average expression ratios of six samples ± SD between the Mp strain and the 410 variant. Supplementary Figure 4: FAMEs and MAMEs were derived from extractable lipids (a) and delipided cells (b) of the Mp and 410 strains. TLC plates were developed in the solvent system η-hexane : ethyl acetate (95 : 5) (thrice) and revealed with CuSO4 and heating. (c) Total extractable lipids of the Mp and 410 strains were analysed by TLC developed in the solvent system chloroform : methanol : water (90 : 10 : 1). [file 4741237.f1.zip › Supplementary figure 1.docx]

## Slide 1
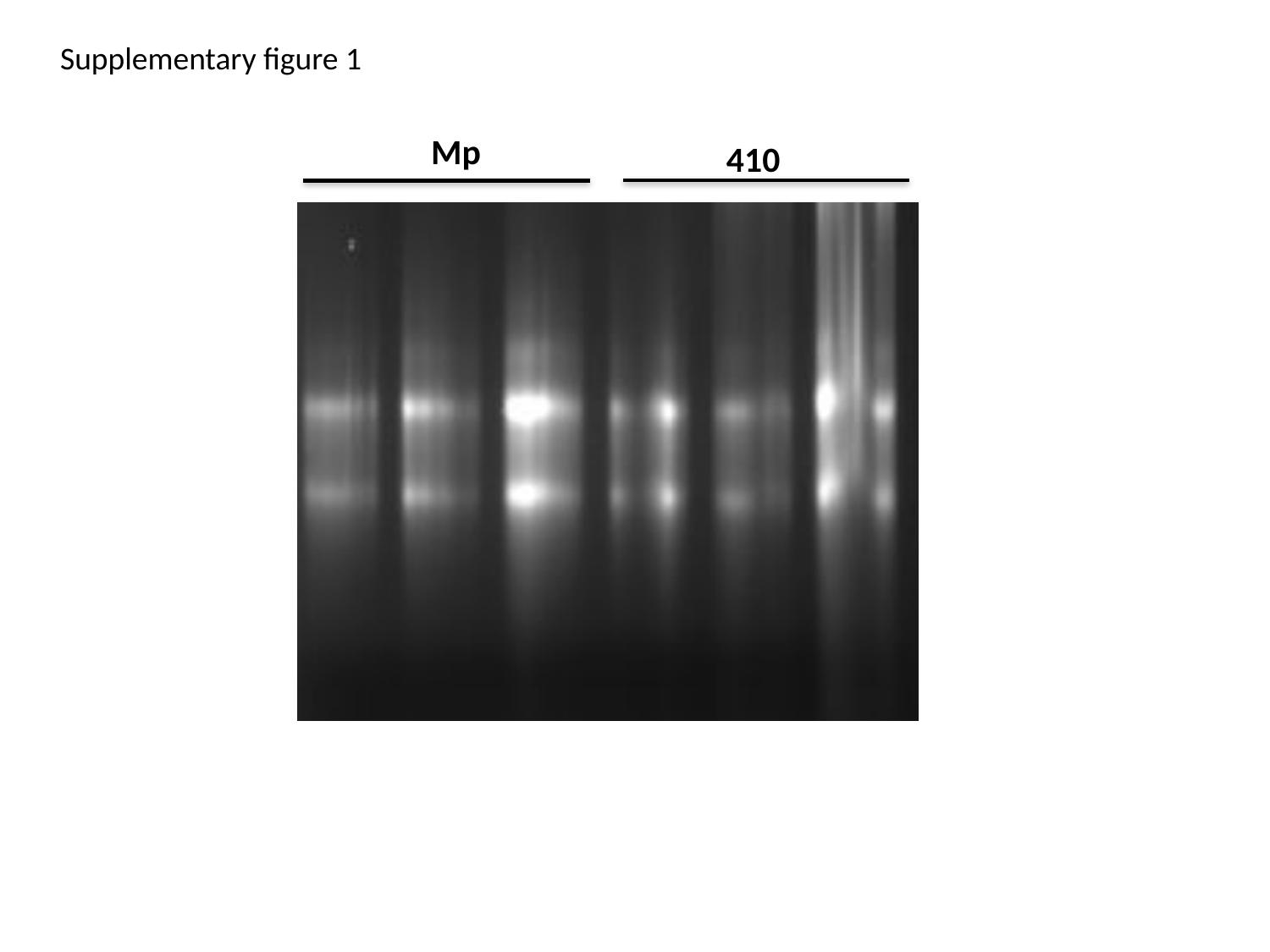

Supplementary figure 1
Mp
410

Supplement: Supplementary Materials — Supplementary Material: Supplementary Table 1, Supplementary Table 2, and Supplementary Table 3. Supplementary Figure 1: (a) chromatogram profile of the high pH reversed-phase (hpRP) fractionation. (b) Schematic representation of the 48 fractions collected in the hpRP chromatography. The labels M1 to M12 in each well indicate the pattern of mixture. Supplementary Figure 2: total RNA obtained from cultures in the stationary phase (OD600nm ~ 1) and resolved in 0.8% agarose gel. Supplementary Figure 3: comparative gene expression of selected DevR regulon genes between strains by RT-qPCR at (a) early stationary and (b) late stationary growth phases. Fold changes of Mp/410 strains were calculated using the expression levels of the sigA mRNA as a reference gene and the 410 variant as a calibrator. Data were analysed using a random permutation test (fg statistic software ∗P < 0.05). The bars represent average expression ratios of six samples ± SD between the Mp strain and the 410 variant. Supplementary Figure 4: FAMEs and MAMEs were derived from extractable lipids (a) and delipided cells (b) of the Mp and 410 strains. TLC plates were developed in the solvent system η-hexane : ethyl acetate (95 : 5) (thrice) and revealed with CuSO4 and heating. (c) Total extractable lipids of the Mp and 410 strains were analysed by TLC developed in the solvent system chloroform : methanol : water (90 : 10 : 1). [file 4741237.f1.zip › Supplementary figure 2.pptx]

## Slide 1
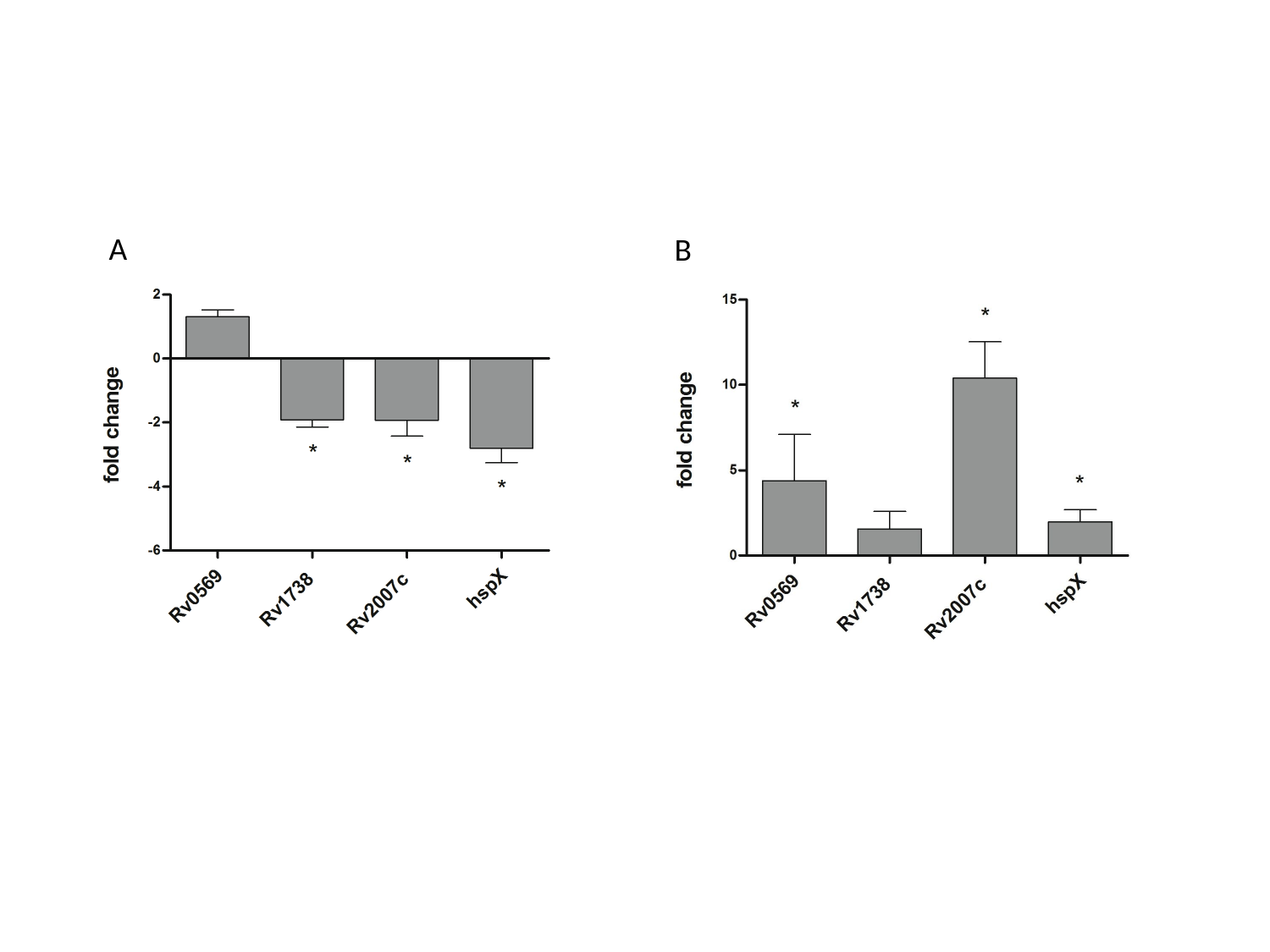

A
B

Supplement: Supplementary Materials — Supplementary Material: Supplementary Table 1, Supplementary Table 2, and Supplementary Table 3. Supplementary Figure 1: (a) chromatogram profile of the high pH reversed-phase (hpRP) fractionation. (b) Schematic representation of the 48 fractions collected in the hpRP chromatography. The labels M1 to M12 in each well indicate the pattern of mixture. Supplementary Figure 2: total RNA obtained from cultures in the stationary phase (OD600nm ~ 1) and resolved in 0.8% agarose gel. Supplementary Figure 3: comparative gene expression of selected DevR regulon genes between strains by RT-qPCR at (a) early stationary and (b) late stationary growth phases. Fold changes of Mp/410 strains were calculated using the expression levels of the sigA mRNA as a reference gene and the 410 variant as a calibrator. Data were analysed using a random permutation test (fg statistic software ∗P < 0.05). The bars represent average expression ratios of six samples ± SD between the Mp strain and the 410 variant. Supplementary Figure 4: FAMEs and MAMEs were derived from extractable lipids (a) and delipided cells (b) of the Mp and 410 strains. TLC plates were developed in the solvent system η-hexane : ethyl acetate (95 : 5) (thrice) and revealed with CuSO4 and heating. (c) Total extractable lipids of the Mp and 410 strains were analysed by TLC developed in the solvent system chloroform : methanol : water (90 : 10 : 1). [file 4741237.f1.zip › Supplementary figure 3.pptx]

## Slide 1
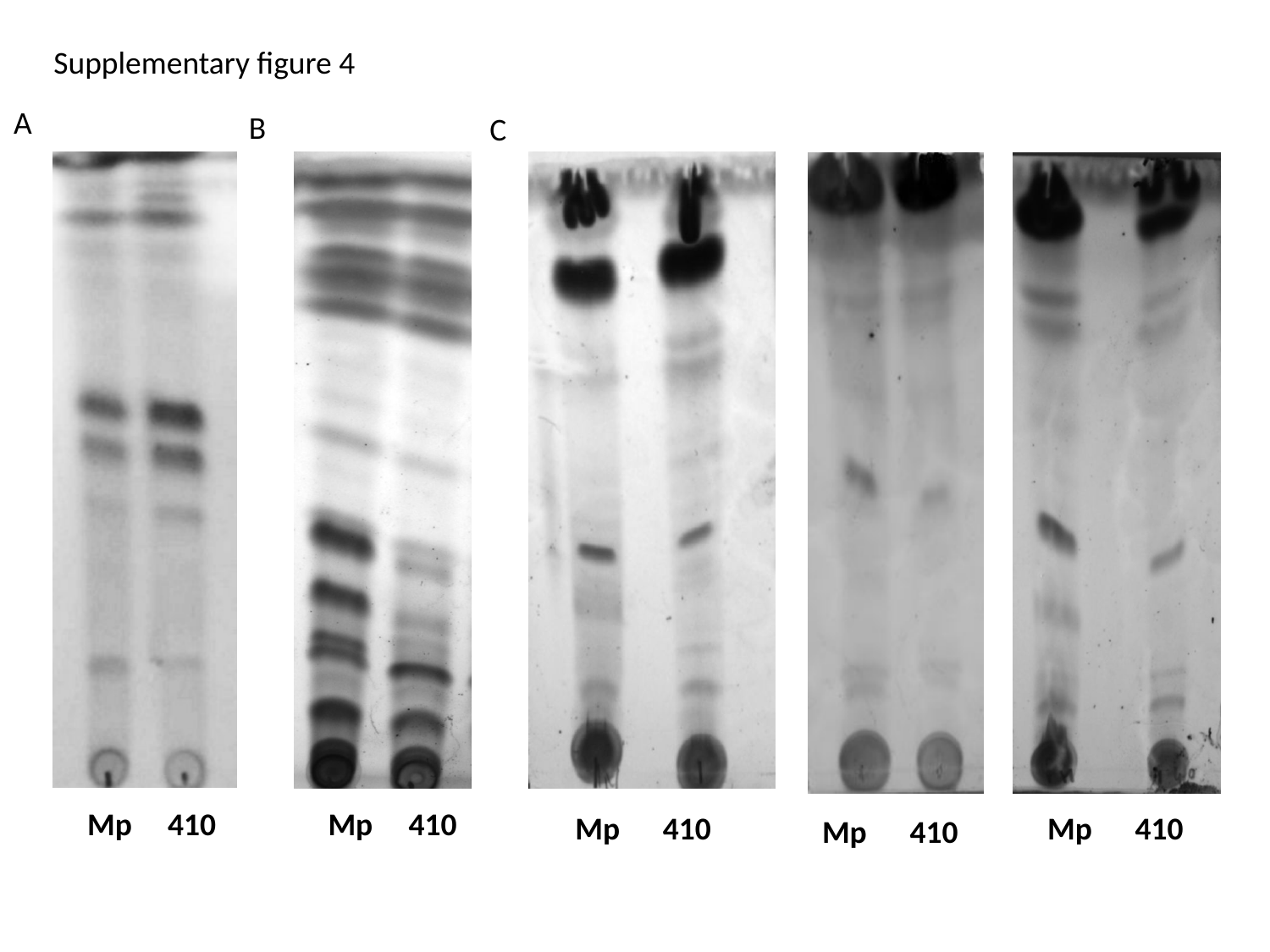

Supplementary figure 4
A
B
C
 Mp 410
 Mp 410
 Mp 410
 Mp 410
 Mp 410

Supplement: Supplementary Materials — Supplementary Material: Supplementary Table 1, Supplementary Table 2, and Supplementary Table 3. Supplementary Figure 1: (a) chromatogram profile of the high pH reversed-phase (hpRP) fractionation. (b) Schematic representation of the 48 fractions collected in the hpRP chromatography. The labels M1 to M12 in each well indicate the pattern of mixture. Supplementary Figure 2: total RNA obtained from cultures in the stationary phase (OD600nm ~ 1) and resolved in 0.8% agarose gel. Supplementary Figure 3: comparative gene expression of selected DevR regulon genes between strains by RT-qPCR at (a) early stationary and (b) late stationary growth phases. Fold changes of Mp/410 strains were calculated using the expression levels of the sigA mRNA as a reference gene and the 410 variant as a calibrator. Data were analysed using a random permutation test (fg statistic software ∗P < 0.05). The bars represent average expression ratios of six samples ± SD between the Mp strain and the 410 variant. Supplementary Figure 4: FAMEs and MAMEs were derived from extractable lipids (a) and delipided cells (b) of the Mp and 410 strains. TLC plates were developed in the solvent system η-hexane : ethyl acetate (95 : 5) (thrice) and revealed with CuSO4 and heating. (c) Total extractable lipids of the Mp and 410 strains were analysed by TLC developed in the solvent system chloroform : methanol : water (90 : 10 : 1). [file 4741237.f1.zip › Supplementary figure 4.pptx]
